# A Case of Neonatal Marfan Syndrome: A Management Conundrum and the Role of a Multidisciplinary Team

**DOI:** 10.1155/2017/8952428

**Published:** 2017-01-11

**Authors:** Elliott J. Carande, Samuel J. Bilton, Satish Adwani

**Affiliations:** ^1^Department of Medical Sciences, University of Oxford, Level 3, John Radcliffe Hospital, Oxford OX3 9DU, UK; ^2^Department of Paediatric Cardiology, John Radcliffe Hospital, Oxford OX3 9DU, UK

## Abstract

Neonatal Marfan syndrome (nMFS) is a rare condition with a poor prognosis. It is genotypically and phenotypically distinct from the typical Marfan syndrome and carries a poorer prognosis. This case report describes the progression of a 14-month-old girl diagnosed with nMFS at 5 months of age. Her diagnosis followed the identification of a fibrillin-1 mutation (*FBN1* gene, exon 26, chromosome 15), which is a common locus of nMFS. This patient developed severe cardiac complications resulting in congestive cardiac failure in early life and required major cardiac surgery. Since surgical intervention, our patient is still reliant on a degree of ventilator support, but the patient has gained weight and echocardiography has demonstrated improved left ventricular function and improved tricuspid and mitral valve regurgitation. Therefore, we argue the importance of a cautious multidisciplinary approach to early surgical intervention in cases of nMFS.

## 1. Introduction

Marfan syndrome is a connective tissue disorder first described by Antoine Marfan in 1896 and is thought to affect 2-3 in 10,000 people [1]. It is inherited in an autosomal dominant fashion and is mostly due to a mutation of the* FBN1* gene on chromosome 15 that encodes the protein fibrillin-1. Marfan syndrome is characterised by disorders of the cardiovascular, musculoskeletal, pulmonary, and ocular systems, as well as the skin [2]. The severity of clinical features varies, and life expectancy in Marfan syndrome is significantly reduced, at 32 ± 16 years for untreated individuals [3], due to their risk of aortic dissection and rupture [1].

Neonatal Marfan syndrome (nMFS) is recognised earlier in life and has more severe clinical features plus a poorer prognosis than the classical Marfan syndrome. In particular, cardiac involvement is more severe in nMFS, with mitral and/or tricuspid valve insufficiency resulting in congestive cardiac failure from a young age [4, 5]. Infantile pulmonary emphysema is also reported more commonly in nMFS, whereas pathology involving the aorta and aortic root is more common in classical Marfan syndrome [4, 5]. Furthermore, joint contractures, megalocornea, iridodonesis, ectopia lentis, redundant loose skin, and crumpled ears have been recognised as more common in the neonatal form.

The prognosis of nMFS is poor. 95% of patients die within the first year of life [6] with data reporting a mean age at death of 16.3 months [3]. However, recent reports have documented patients with nMFS at 4 and 11 years of age [7, 8]. Early diagnosis of the condition and the initiation of treatment are essential to prevent the development of the refractory heart failure [7], the cause of death in 85% of patients with nMFS [9].

Marfan syndrome is caused by mutations in the* FBN1* gene on chromosome 15, which encodes the protein fibrillin-1. These mutations spread out over the whole gene. Mutations causing nMFS also affect the* FBN1* gene but these de novo mutations consistently cluster in exons 23–32 of the gene [7], in what is regarded as one of the few accepted genotype-phenotype correlations described to date [10]. nMFS may arise due to mutations outside this region, although this has only been reported three times in the literature, in exon 4 twice and in exon 21 once [9, 11]. Similarly, mutations in exons 23–32 of the* FBN1* gene may also lead to classical Marfan syndrome. Mutations in exons 25-26 are overrepresented and are associated with shorter survival in children diagnosed with* FBN1* mutations before the age of 1 year [9].

In this case study we report the early life, diagnosis, and management of a child with nMFS who suffered severe cardiac failure with mitral and tricuspid valve regurgitation, and aortic root dilatation, alongside global developmental delay. The child underwent surgery at 11 months and is now 14 months of age.

## 2. Case Presentation

This 11-month-old girl is the first child of healthy nonconsanguineous parents, neither having a Marfan diagnosis (although not formally tested). There is no other family history of Marfan disease, aortic disease, or sudden death.

### 2.1. Pregnancy and Birth

The pregnancy was planned. Anomaly scans at 33 weeks showed increased leg length, and repeat scans at 37 weeks demonstrated oligohydramnios. This resulted in steroid induction at 37 + 2 weeks. After birth, she did not require any resuscitation or special care but was found to have positional talipes. At birth, her length was 49.6 cm (>75th centile) and head circumference 33 cm (50th centile). Her birth weight was 2.335 kg (9th centile).

### 2.2. Early Life

**Figure 1:**
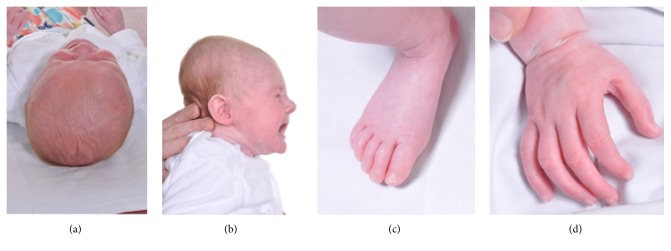
Photos of the proband taken at 6 weeks: (a) plagiocephaly with prominent coronal sutures. (b) Turricephaly. (c, d) Arachnodactyly. © Oxford Medical Illustration.

She was referred to a genetics clinic at 6 weeks due to concern over her physical features. On examination, her head circumference was 39 cm (99.6th centile) and length was 55 cm (75th–91st centile). Her head was plagiocephalic with prominent coronal sutures and a posteriorly positioned anterior fontanelle, and her posterior skull was turricephalic with a cone shape (see Figure 1). Her zygomatic arches were prominent.

Her fingers and toes appeared elongated (arachnodactyly), although lengths were not objectively measured, and her thumb held abducted (see Figure 1). She had clicky joints in her legs and was unable to fully extend her knees. Her feet bent up so that the top of her foot touched her shin. She had kyphoscoliosis.

There was presence of a divergent squint, although eye exam was otherwise unremarkable, with no lenticular dislocation nor iridodonesis noted. Her palate was normal. Her right nipple was positioned more laterally compared to the left and her ribs were more prominent on the right. The remainder of the clinical examination was unremarkable.

### 2.3. Molecular Studies and Echocardiography

**Figure 2:**
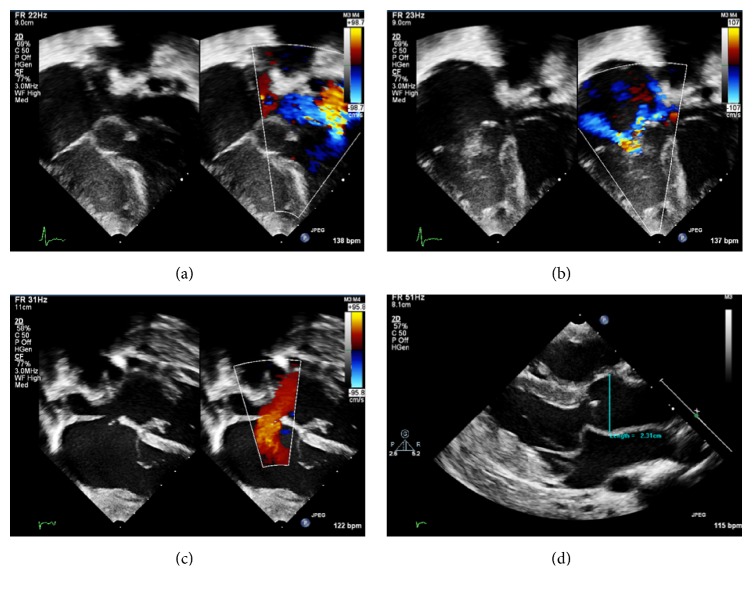
Presurgical echocardiographic features of the proband: (a) apical 4-chamber view demonstrating mitral regurgitation. (b) Apical 4-chamber view demonstrating tricuspid regurgitation. (c) Subcostal 4-chamber view demonstrating atrial septal defect. (d) Parasternal long axis view demonstrating mitral valve prolapse and aortic root dilatation.

In light of the dysmorphic features identified, genetic investigations were carried out on suspicion of nMFS. There was confirmation of an* FBN1* gene mutation (exon 26, c.3143T>C). Echocardiography was performed which showed aortic root dilatation at 5 months of age, with a maximum diameter of 18 mm (Z + 4.3). In addition, echocardiography demonstrated atrioventricular valve prolapse, tricuspid regurgitation, a dysplastic mitral valve with mitral regurgitation, an increase in right ventricular pressure, and pulmonary hypertension (see Figure 2).

### 2.4. Hospital Admissions

At 5 months of age, she was admitted due to signs of heart failure and respiratory distress, with a click and pansystolic murmur of mitral regurgitation on cardiac auscultation. She was started on medical heart failure therapy (captopril, spironolactone, and furosemide) and was discharged home following improvement. The child was started on captopril (an ACE inhibitor) instead of an alternative angiotensin receptor blocker, as this was consistent with local trust guidelines.

Worsening respiratory function at 7 months required further admission to rule out bronchiolitis and pneumonia, although no formal lung function tests were carried out. Our patient was commenced on sildenafil in light of the clinical picture and previous echocardiographic demonstration of pulmonary hypertension.

**Figure 3:**
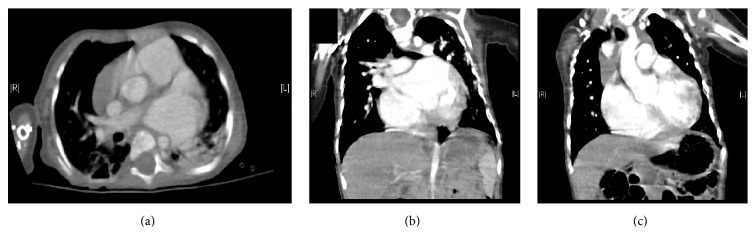
CT scan taken at 7 months: (a) enlarged left atrium and dilated pulmonary trunk. (b) Atrial septal defect. (c) Dilated aortic root and patent ductus arteriosus.

Echocardiogram revealed worsening regurgitation, dilated pulmonary arteries, and pulmonary hypertension and a CT scan additionally demonstrated right sided cardiac enlargement, an atrial septal defect, and a patent ductus arteriosus (see Figure 3). At 8 months of age she required intubation and mechanical ventilation for further deteriorations. Our patient was started on the phosphodiesterase-3 inhibitor milrinone, and captopril was converted to irbesartan on the advice of the intensive care unit. Since then, she remained on mechanical ventilation due to a combination of cardiac failure and a restrictive lung physiology related to scoliosis and severe hypotonia. Digoxin was started one month later due to persistent cardiac failure.

### 2.5. Cardiac Surgery

A multidisciplinary team (MDT) meeting discussed the feasibility of cardiac surgery given the severity of congestive cardiac failure, and it was concluded that surgery would not be in the patient's best interests. The nMFS presentation, severe hypotonia, and its impact on early and late postoperative morbidity were the main concern, with the comorbidities preventing a good long-term outcome.

A second opinion concerning cardiac surgery was requested by the parents. A clinical geneticist's view was sought and they emphasised that the main prognosis for this child was dependent upon her life limiting cardiovascular status and that disease related complications would be manageable after surgery and compatible with a good quality of life. Consequently, it was decided that cardiac surgery would be in the patient's best interests. It is important to note that this view was subjective but discussed and agreed on by an MDT including cardiologists and cardiovascular surgeons. In preparation for surgery, and in light of worsening cardiovascular and respiratory function, the patient was given a tracheostomy to facilitate long-term ventilation.

The patient underwent tricuspid valve repair (leaflet repair and partial annuloplasty), mitral valve repair (leaflet chordae shortening and partial annuloplasty), and ASD closure at 11 months of age. Direct inspection of her lungs showed very significant pulmonary emphysema. The surgery was uneventful with no bleeding and no rhythm issues apart from slow sinus rhythm. She was admitted to PICU in a stable condition. Nasogastric feeding was resumed 2 days after surgery.

**Figure 4:**
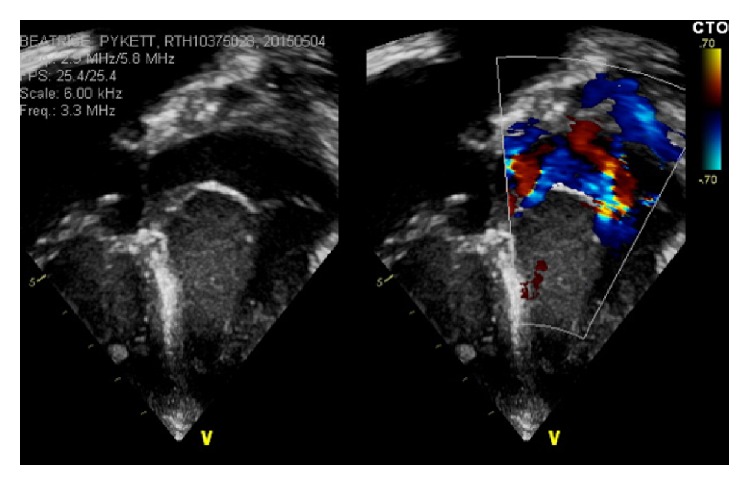
Postsurgical echocardiographic features of the proband: apical 4-chamber view demonstrating reduced mitral regurgitation.

From a cardiovascular point of view, the patient remained stable following the surgery. She required inotropic support with adrenaline for 2 hours and required pacing via epicardial pacemaker for 24 hours due to slow sinus rhythm. A postoperative transthoracic echo in PICU confirmed good surgical results with good biventricular function, mild mitral regurgitation, and mild-moderate tricuspid regurgitation (see Figure 4).

### 2.6. Postoperative Progress

Following surgery, the patient remains ventilated through a tracheostomy but has had gradually increasing periods off ventilator (30 minutes–1 hour every 2-3 hours). She developed frequent daily vomiting due to gastrooesophageal reflux disease, which was treated with omeprazole. Due to feeding difficulties, a PEG was inserted. Since then she has been progressing well and is gaining weight. Immediately before operation, at 11 months of age, the patient weighed 5.80 kg (<0.4th centile). At 14 months of age she weighed 8 kg (2nd–9th centile).

## 3. Discussion

Clinically, neonatal Marfan syndrome differs from the presentation of classic Marfan syndrome in infants through the severity of cardiac and pulmonary manifestations, in particular mitral valve prolapse, mitral, tricuspid, and pulmonary regurgitations and congenital pulmonary emphysema [12]. Consistent with this, from 5 months of age, our patient developed signs of heart failure and respiratory distress with echocardiography confirming mitral valve prolapse with regurgitation alongside tricuspid valve prolapse with regurgitation and a dilated aortic root.

In addition, echocardiography demonstrated an ASD and PDA in our patient, an unusual finding in nMFS, and more characteristic of the related Loeys-Dietz syndrome [13]. From our research of the literature, we have found two other cases of ASD in nMFS [14]. Interestingly, these septal defects were only demonstrated at postmortem of the cases and were not mentioned on echocardiography findings. Three further cases of patent foramen ovale have been also been found, two in the same study at postmortem [14] and one further case on echocardiography in a 4-month-old boy [15]. No evidence of PDA in nMFS was found in the literature.

One of the pathogenic mechanisms of nMFS is thought to be a paradoxical increase in TGF-*β* caused by abnormal fibrillin-1 activity, demonstrated by an increased level of TGF-*β* in the aortic wall of fibrillin-1 deficient mice [16]. TGF-*β* is thought to play a role in the proliferation of vascular smooth muscle cells and may result in aortic root dilatation [16]. Excess TGF-*β* signalling has also been shown to play a role in other aortic aneurysm syndromes, including Loeys-Dietz syndrome [13, 17].

Angiotensin II receptor type 1 (AT1) activation also increases the production of TGF-*β* [18] and therefore selective inhibition of the AT1 receptor offers a therapeutic target to favourably modify the pathogenesis of tissue injury in nMFS. Our patient was started on irbesartan, an AT1 receptor blocker, at 8 months of age on the advice of a specialist intensive care unit. Habashi et al. used a mouse model of MFS to demonstrate that aortic aneurysm in mice can be prevented by the AT1 blocker, losartan, the effects of which were greater than the effects of beta blockade [18]. Furthermore, they demonstrated that AT1 antagonism could reverse some noncardiovascular clinical features of MFS, including impaired alveolar septation. This has been backed up by evidence that infusion of angiotensin II causes increased aortic dilatation in mice [19, 20].

Experiments on human Marfan patients, however, have yielded inconclusive results in comparison to mice models. In a prospective randomized trial [21], losartan use showed no benefit compared to beta blockade, whilst in a separate trial angiotensin receptor blockers slowed the rate of aortic dilatation after all other medical therapies had failed [22]. Further human studies are awaited, including AIMS (Aortic Irbesartan Marfan Study) [23], a prospective, randomized, placebo-controlled, double-blind, multicenter study of the effects of irbesartan on aortic root dilatation in 490 patients with Marfan syndrome, with results expected in 2018-2019. There have been case reports of the potential effects of AT1 antagonists in nMFS [24], but no randomized control studies have, as yet, reported the efficacy of these medications in this group.

Early recognition of nMFS is vital to allow for attempted treatment planning and prognosis modification. Valvular insufficiencies, congestive heart failure, and/or aortic dissection are the severe manifestations of nMFS [3] and, therefore, surgical intervention should be considered early to prevent mortality in this group of patients, as medical treatment is usually unable to control heart failure symptoms in these patients [3]. Both recent reports of improved survival in nMFS have been in patients with corrective cardiac surgery [7, 8]. However, given the small number of reported cases, it is entirely plausible that cases with poor outcomes from surgical intervention may be underreported in the literature.

Heart surgery in patients with nMFS is complex and carries with it the risk of mortality and morbidity, including heart block, thrombosis, and stroke [3]. It is important that these decisions are taken with a multidisciplinary approach, with paediatric cardiologist, cardiothoracic surgeon, geneticist, and nursing input, to determine whether invasive, life-threatening surgery is in the best interests of the patient.

Our patient is still young and has had a relatively short postoperative follow-up period. Thus our patient's prognosis is unclear, which is especially difficult to predict given the preoperative treatment requirements and comorbidities (including tracheostomy with continued BIPAP support, PEG feeding, and severe hypotonia). It remains to be seen how our patient will respond with time but our patient is gaining weight much more rapidly than before surgery and has decreasing ventilator requirements, and the current indications are that surgery has reduced signs of congestive cardiac failure, hence improving prognosis.

### 3.1. Conclusion

The major cause of death in nMFS is from congestive cardiac failure, which develops early in life. Evidence from the literature suggests that early cardiac surgery can significantly improve symptoms and prognosis. From our case report, we advocate the importance of a multidisciplinary approach to cases of nMFS when considering a treatment plan and stress that early surgical management should be seriously considered in children with nMFS, whilst taking comorbidities into account. It is too soon to know how effective cardiac surgery has been when considering the long-term prognosis of our patient, but we hope that such surgery will prolong the patient's life, allowing more time for the long-term potential beneficial effects of intensive medical management.
